# NRBP2 Functions as a Tumor Suppressor and Inhibits Epithelial-to-Mesenchymal Transition in Breast Cancer

**DOI:** 10.3389/fonc.2021.634026

**Published:** 2021-03-18

**Authors:** Zhiyu Li, Bingxiong Liu, Chenyuan Li, Si Sun, Hanpu Zhang, Shengrong Sun, Zhong Wang, Xiongjie Zhang

**Affiliations:** ^1^Department of Breast and Thyroid Surgery, Renmin Hospital of Wuhan University, Wuhan, China; ^2^Department of General Surgery, Hanchuan People's Hospital, Hanchuan, China; ^3^Department of Clinical Laboratory, Renmin Hospital of Wuhan University, Wuhan, China

**Keywords:** NRBP2, breast cancer, cell survival, EMT, AMPK/mTOR pathway

## Abstract

Nuclear Receptor Binding Protein 2 (NRBP2), one of the pseudokinases discovered during a screen of neural differentiation genes, inhibits tumor progression in medulloblastoma and hepatocellular carcinoma. However, the role and the mechanism of NRBP2 in the regulation of the progression of breast cancer (BC) have not been reported. In our study, NRBP2 was downregulated in human BC tissues compared with the corresponding normal tissues. Moreover, bioinformatics and cellular experiments illustrated that a lower level of NRBP2 contributed to a poor prognosis for patients with BC. In addition, we characterized the NRBP2-overexpressing BC cells and found that NRBP2 overexpression dramatically suppressed cell proliferation and invasion and inhibited the epithelial-mesenchymal transition (EMT) in cells *in vitro*, whereas knockdown of NRBP2 reversed these effects. Furthermore, overexpression of NRBP2 in the orthotopic breast tumor model significantly reduced lung metastatic nodules in nude mice. Mechanistically, NRBP2 regulated the activation of the 5′-adenosine monophosphate (AMP)-activated protein kinase/ mammalian target of rapamycin (AMPK/mTOR) signaling pathway. Moreover, the inhibition of cell proliferation, invasion and the EMT by NRBP2 overexpression was partially rescued after treatment with an AMPK inhibitor. Conversely, mTOR-specific inhibitors eliminated the effects of NRBP2 knockdown on increasing cell proliferation, invasion and the EMT, which suggested the anti-tumor effect of NRBP2, which may be partially related to the regulation of the AMPK/mTOR pathway. Taken together, NRBP2, a novel and effective prognostic indicator, inhibited the progression of BC and may become a potential therapeutic target for BC.

## Introduction

Breast cancer (BC) is the most prevalent neoplasm among women and is the leading cause of cancer-related death in women. BC-related deaths account for 6.6% of all cancer-related deaths (1). Currently, surgery, radiation treatment, targeted endocrine therapy and chemotherapy are the most common therapeutic approaches. However, distant metastases remain a major problem.

In the invasive and metastatic phenotype, breast carcinoma cells exhibit fewer epithelial features and a greater number of mesenchymal traits, which is the process termed the epithelial-to-mesenchymal transition (EMT) (2). During the EMT, cell polarity and cell adhesion are lost by removing tight junction proteins such as E-cadherin from the cell surface and upregulating the expression of mesenchymal proteins such as N-cadherin (3). Thus, the epithelial structural support is impaired, cell motility is increased and new membrane protrusions are formed, which facilitate cell migration and invasion (4).

The 5′-adenosine monophosphate (AMP)-activated protein kinase (AMPK) signaling pathway is reported to inhibit the EMT in pancreatic cancer (5), hepatocellular carcinoma (HCC) (6), and breast cancer (7). AMPK is activated in response to various stresses, including nutrient deprivation and hypoxia. It is involved in several physiological processes, such as energy homeostasis, cell growth and autophagy. Mammalian target of rapamycin (mTOR) is one of the main targets of AMPK and is inhibited by AMPK activation. The AMPK/mTOR axis is able to modulate some EMT-related transcription factors, including twist and snail, thus mediating the EMT (8).

Nuclear Receptor Binding Protein 2 (NRBP2) is a 55–60 kDa conserved protein (9) with a cytoplasmic localization in neural stem/progenitor cells (NSPCs) (9), brain tumor cells (10) and HCC cells (11). The NRBP family is initially involved in the transport between the endoplasmic reticulum and Golgi (12). During tumor progression, NRBP2 is consistently regarded as a tumor suppressor gene (13). NRBP2 causes the death of NSPCs, medulloblastoma cells and HCC cells (9–11). It also positively regulates the cytotoxic effects of chemotherapy in HCC (11).

In our study, we investigated the function of NRBP2 in BC and further explored the molecular mechanism by which NRBP2 mediated the EMT. This study aimed to provide new potential therapeutic targets for patients with BC.

## Materials and Methods

### Data Acquisition

The expression of NRBP2 and its relationship with the prognosis of patients with different types of BC were analyzed using the UALCAN (http://ualcan.path.uab.edu), UCSC Xena (https://xena.ucsc.edu/), GEPIA (http://gepia.cancer-pku.cn/) databases and Kaplan-Meier Plotter (https://kmplot.com/).

### Reagents and Antibodies

Antibodies against E-cadherin (Santa Cruz, sc-7870), N-cadherin (Cell Signaling Technology, 13116), Snail (Santa Cruz, sc-393172), NRBP2 (Proteintech, 21549-1-AP), p-AMPK (Cell Signaling Technology, 2535), AMPK (Cell Signaling Technology, 5831), p-mTOR (Cell Signaling Technology, 5536), mTOR (Cell Signaling Technology, 2983), and actin (Sigma, A5441) were used. Compound C and rapamycin were purchased from MedChemExpress and prepared according to the manufacturer's instructions.

### Cell Culture

Human BC cell lines (MCF7 and MDA-MB-231) were purchased from American Type Culture Collection (ATCC, Rockville, MD). All cell lines were verified by STR spectrum. All cell lines were cultured in DMEM (Gibco, USA) containing 10% FBS at 37°C with 5% CO_2_.

### Cell Transfection

Flag-NRBP2 and Flag-NC plasmids were purchased from GeneChem Co. (Shanghai, China). The NRBP2 siRNA (1# 5′-GGAGAUGGCUGUACUGGAATT; 2# 5′-GGUACUCGGAAGUCUCCUUTT) and scrambled siRNA (5′-UUCUCCGAACGUGUCACGUTT) were synthesized by GenePharma Co. (Shanghai, China). An appropriate number of cells was seeded in six-well plates, and Lipofectamine 3000 (Invitrogen, USA) was used to transfect the plasmid and siRNA into the cells as described in a previous study (14) and according to the supplier's instructions.

### Immunohistochemical Staining

IHC staining was performed using the method reported in a previous study (14). The evaluation and scoring methods were described in our previous study (14). The immunohistochemical score results were independently completed by two pathologists. The scoring criteria for the percentage of positive cells was as follows: 0, <10%; 1, 10–30%; 2, 31–50%; 3, >50%. The staining intensity was evaluated as follows: 0 = no expression, 1 = weak intensity, 2 = moderate intensity, 3 = strong intensity. The final score was intensity score multiplied by the percentage of positive cells score. All samples were divided into three levels: 0 = negative, 1–3 = low expression and 4–9 = high expression.

### Cell Counting Kit-8 Assay

An appropriate number of cells was plated in 96-well plates and the absorbance value of cells cultured for different days was measured using the CCK-8 assay (CK04, Dojindo, Japan) according to the manufacturer's instructions.

### Invasion Assay

After various treatments, 4 × 10^4^ cells were plated in the upper chambers coated with Matrigel (Corning, NY, USA). Medium in the upper chambers lacked serum. Medium in the bottom chambers contained 10% FBS. Crystal violet (C0121, Beyotime) was used to stain the cells. The protocol was described in a previous study (14).

### Western Blotting

The protocol was described previously (14). The results of western blotting were obtained by Odyssey Infrared Imager (Li-COR Biosciences, USA). The expression levels of proteins were quantified using ImageJ software (W S Rasband, Image J, NIH).

### Animal Experiments

MDA-MB-231 cells were transfected with the NRBP2 overexpression lentivirus purchased from GeneChem Co. (Shanghai, China) and selected with puromycin. Western blotting was used to detect the level of NRBP2. All experimental procedures were conducted according to the Regulations of Experimental Animal Administration issued by Animal Committee of Wuhan University. Xenograft models were established in 4- to 5-week-old female athymic nude mice (BALB/c) purchased from The Animal Center of Renmin Hospital of Wuhan University. Eight mice were randomly divided into two groups, and 1.5 × 10^6^ stable cells were resuspended in PBS and Matrigel mixed in equal proportions and injected into the fourth mammary fat pad. Tumors were excised when their size reached approximately 300 mm^3^. These mice were sacrificed under anesthesia at 10 days after the operation, and then the number of metastatic tumors in the lung were determined. The entire lung tissues were fixed with 10% formalin. Haematoxylin and eosin (HE) staining was performed to determine the presence of lung metastasis.

### Statistical Analysis

The data were analyzed using SPSS 24.0 (SPSS Inc., Chicago, IL) and the graphs were prepared using GraphPad Prism 8 software (La Jolla, CA, USA). All experiments were repeated at least three times and the data are presented as the means ± SD. *P* < 0.05 was considered statistically significant.

## Results

### NRBP2 Is Downregulated in BC Tissues and Positively Associated With the Prognosis of Patients With BC

**Figure 1:**
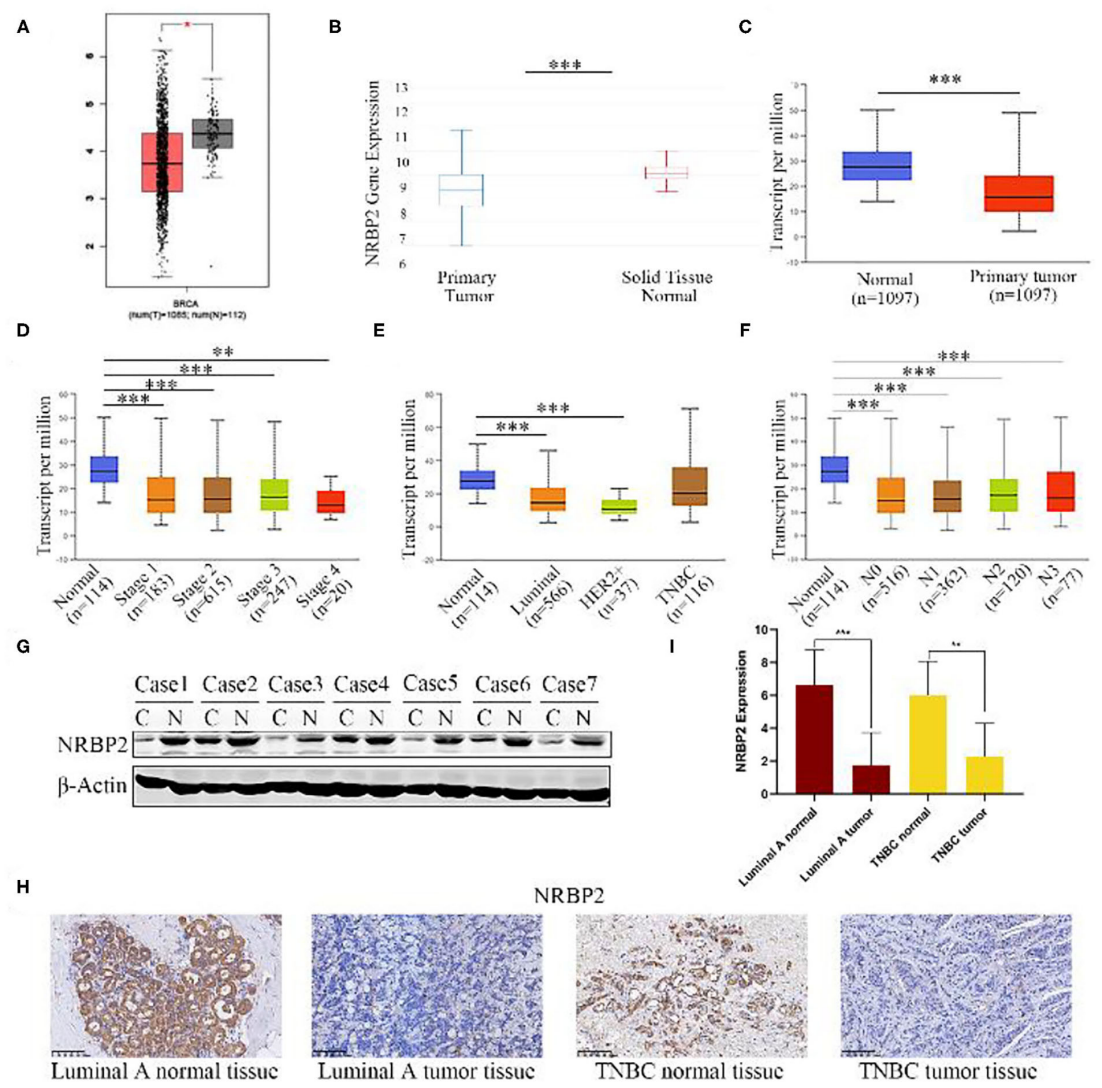
NRBP2 expression is downregulated in BC tissues. **(A–C)** Relative levels of NRBP2 in BC tissues and adjacent tissues in TCGA database from GEPIA, UCSC Xena and UALCAN. **(D)** NRBP2 expression in different stages of BC (UALCAN). **(E)** NRBP2 expression in different subtypes of BC (UALCAN). **(F)** NRBP2 expression in different lymph node metastasis stages of BC (UALCAN). **(G)** Levels of the NRBP2 protein in BC tissues and adjacent tissues. **(H,I)** Immunohistochemical staining for NRBP2 in BC tissues and adjacent tissues (magnification ×200). Quantitative analysis is shown in **(I)**. ^*^p < 0.05 and ^***^p < 0.001 compared with the control group.

We first analyzed the levels of NRBP2 expression in non-tumor breast tissues and BC tissues from three different websites (http://ualcan.path.uab.edu, https://xena.ucsc.edu/, and http://gepia.cancer-pku.cn/) to explore the role of NRBP2 in BC. Data from the three different websites were consistent with the conclusion that NRBP2 is expressed at significantly higher levels in normal tissues than in tumor tissues (Figures 1A–C). As shown in Figures 1D,F, the levels of NRBP2 transcripts were negatively associated with BC stages and lymphatic metastasis stages. Moreover, significantly lower levels of NRBP2 expression were observed in different subtypes of BC than in the normal tissue (Figure 1E). Then, we detected NRBP2 expression levels in BC tissues and normal tissues from patients with BC treated at Renmin Hospital of Wuhan University. As shown in Figures 1G–I, the expression of NRBP2 detected using Western blotting and IHC was significantly increased in normal tissues compared to BC tissues.

**Figure 2:**
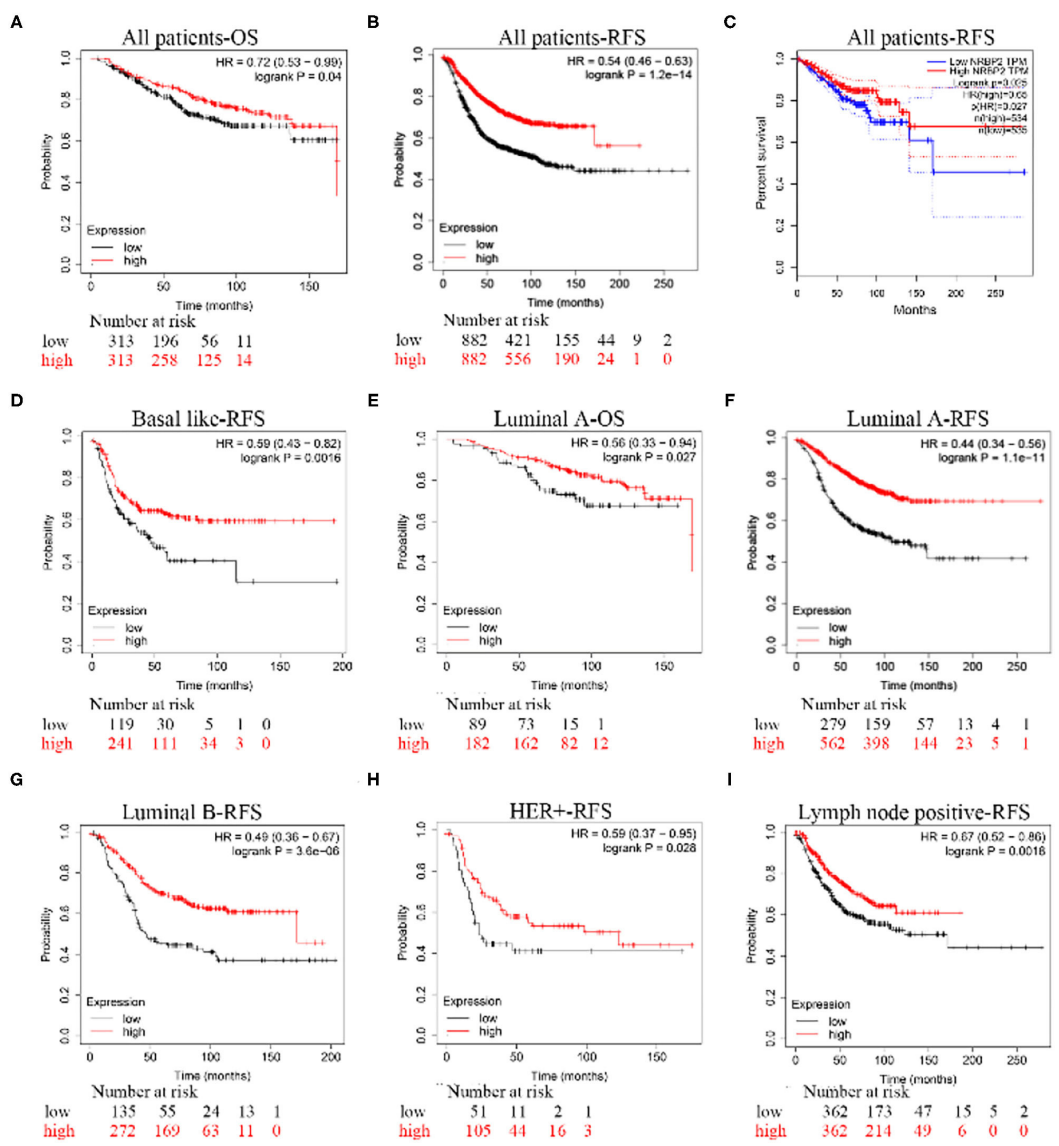
A poor prognosis of patients with BC presenting low NRBP2 expression. The K-M analysis of the **(A)** OS curve and **(B)** RFS curve for all patients created using Kaplan-Meier Plotter. **(C)** The RFS curve for all patients was derived from GEPIA. **(D)** The RFS curve for patients with basal-like BC, **(E)** OS curve for patients with Luminal A BC, **(F)** RFS curve for patients with Luminal A BC, **(G)** RFS curve for patients with Luminal B BC, **(H)** RFS curve for patients with HER2-positive BC and **(I)** RFS curve for patients with lymph node-positive BC were all created using Kaplan-Meier Plotter.

The Kaplan–Meier analysis of recurrence-free survival (RFS) and overall survival (OS) in all patients and patients with the Luminal A subtype of BC showed that the group with high NRBP2 expression had a better outcome than the group with low NRBP2 expression (Figures 2A–C,E,F). Moreover, the analysis of the RFS of patients with other subtypes of BC, such as basal like, Luminal B, Her+ and lymph node-positive, also produced similar results as the analysis of all patients and patients with the Luminal A subtype of BC (Figures 2D,G–I). Based on these results, NRBP2 expression was downregulated in BC tumor tissues, and the decrease in NRBP2 expression might be a factor correlated with a poor prognosis of BC.

### Overexpression of NRBP2 Inhibits Cell Proliferation and Invasion *in vitro*

**Figure 3:**
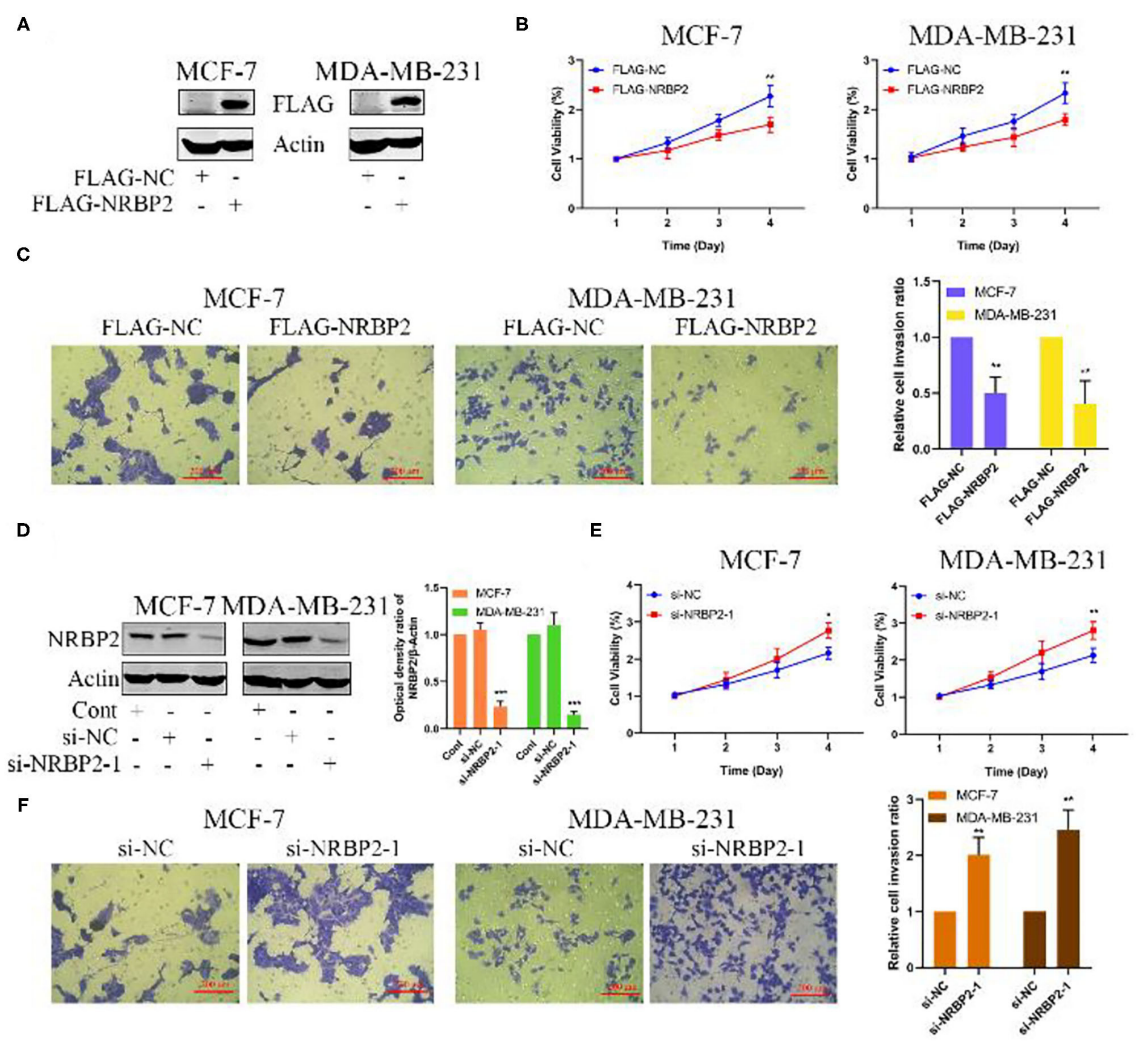
Overexpression of NRBP2 inhibits cell proliferation and invasion *in vitro*. **(A)** The transfection efficiency of NRBP2 in MCF7 and MDA-MB-231 cells. **(B)** Cell viability was measured using the CCK-8 assay after NRBP2 overexpression in two BC lines. **(C)** The invasion of BC cells overexpressing NRBP2 was confirmed using the Transwell assay. The results from the quantitative analysis of the percentage of invading cells are shown (magnification ×200). **(D)** The knockdown efficiency of NRBP2 in BC cell lines. **(E)** Cell growth was measured using the CCK-8 assay after NRBP2 knockdown in two BC lines. **(F)** The invasion of BC cells with knockdown of NRBP2 was determined using Transwell assay. The results from the quantitative analysis the percentage of invading cells are shown (magnification ×200). The values are presented as the means ± SD from three independent experiments. ^*^p < 0.05, ^**^p < 0.01, and ^***^p < 0.001 compared with the control group.

We transfected the FLAG-NRBP2 plasmid into MCF7 and MDA-MB-231 cells to overexpress the NRBP2 protein and further explore its effect on BC growth and invasion; with Flag-NC was used as a control for comparison. As shown in Figure 3A, NRBP2 was significantly overexpressed in BC cell lines. Cell viability decreased after NRBP2 overexpression in both BC cell lines compared with the control group (Figure 3B). In addition, MCF-7 and MDA-MB-231 cells overexpressing NRBP2 exhibited a slower rate of invasion in collagen gels than the control cells (Figure 3C). Furthermore, we used siRNA oligonucleotides specifically targeting NRBP2 to downregulate its expression in BC cell lines. The knockdown efficiency is shown in Figure 3D and Supplementary Figure 1A. The same experiments were performed again to assess the proliferation and invasion. As shown in Figures 3E,F and Supplementary Figures 1B,C, the cell viability and percentage of invading cells both increased after NRBP2 knockdown. Taken together, overexpression of NRBP2 in BC cell lines inhibited cell proliferation and invasion.

### Overexpression of NRBP2 Inhibits the EMT in BC Cells *in vitro*

**Figure 4:**
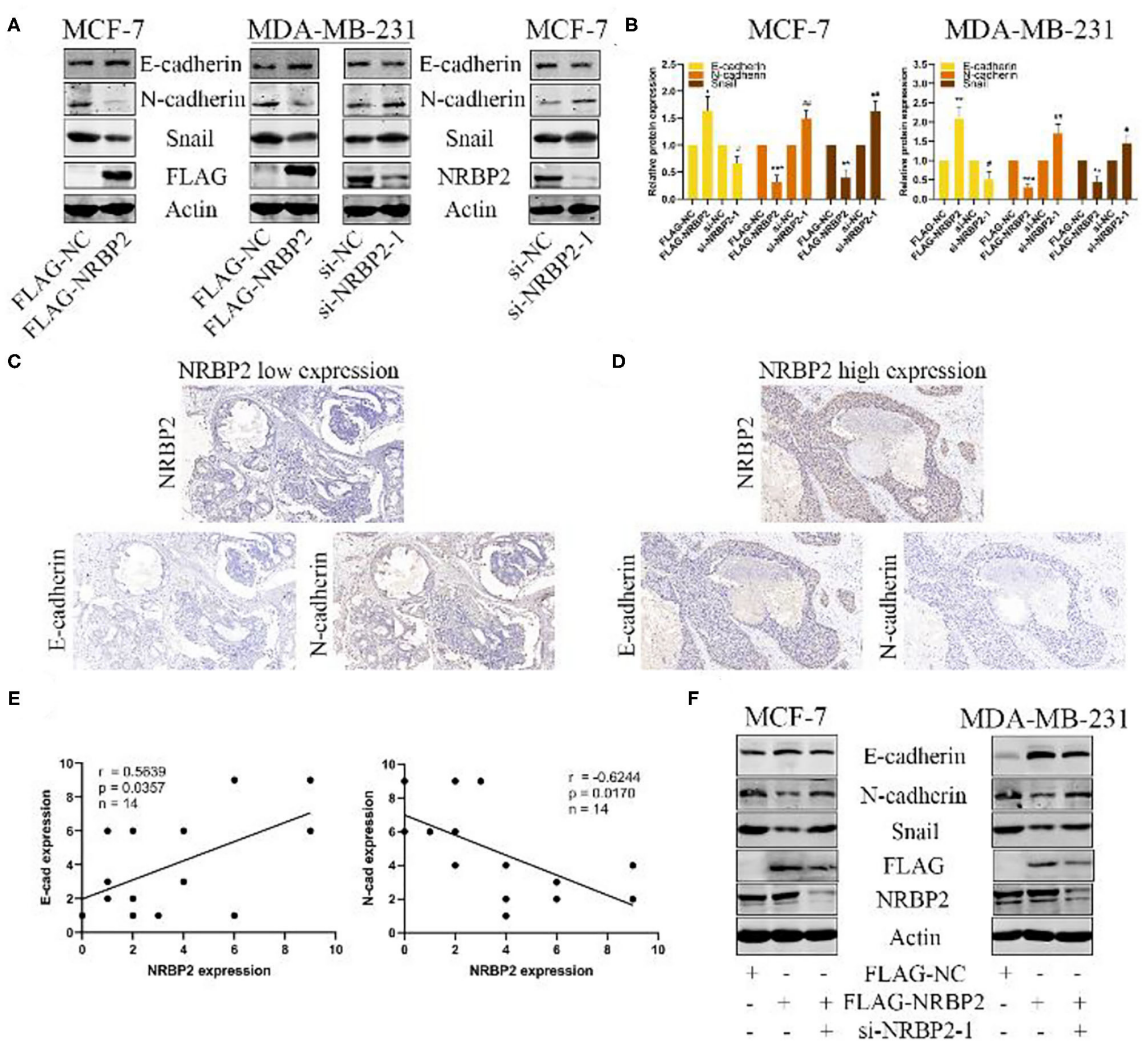
Overexpression of NRBP2 inhibits the EMT in BC cells *in vitro*. **(A)** Levels of the EMT-related proteins E-cadherin, N-cadherin and Snail in NRBP2-knockdown and -overexpressing cells were detected using Western blotting. **(B)** Quantitative analysis of the optical density ratio of E-cadherin, N-cadherin and Snail levels normalized to β-Actin levels. **(C,D)** Images of IHC staining for NRBP2, E-cadherin and N-cadherin in BC tissues (magnification ×200). **(E)** Correlation analysis for of NRBP2 and E-cadherin/N-cadherin. **(F)** NRBP2 was knocked down in NRBP2-overexpressing cells. Western blotting was performed to detect the levels of EMT-related proteins. The values are presented as the means ± SD from three independent experiments. ^*^p < 0.05, ^**^p < 0.01 and ^***^p < 0.001 compared with the control group.

We measured the protein levels of EMT related markers, such as E-cadherin, N-cadherin and Snail, both in NRBP2-overexpressing and -knockdown BC cell lines to elucidate the mechanism by which NRBP2 represses cell proliferation and invasion. As shown in Figures 4A,B and Supplementary Figure 1D, the level of the epithelial marker E-cadherin was increased in NRBP2-overexpressing cells and decreased in NRBP2-knockdown cells. In the contrast, the expression of the mesenchymal markers N-cadherin and Snail was decreased after NRBP2 overexpression and increased after NRBP2 knockdown. Alternatively, we also performed IHC staining of breast tumor tissues with antibodies against NRBP2, E-cadherin and N-cadherin. The expression of NRBP2 was positively correlated with E-cadherin expression and negatively with N-cadherin expression (Figures 4C,D). The correlation analysis of NRBP2 and E-cadherin/N-cadherin was shown in Figure 4E. Next, a rescue experiment was performed to further verify our results. We transfected Flag-NRBP2 into MCF7 and MDA-MB-231 cells, and then si-NRBP2 was transfected into these cells to inhibit NRBP2 expression. Protein levels of EMT-related markers were detected using Western blotting. As shown Figure 4F, knock down of NRBP2 in NRBP2-overexpressing cells partially restored the levels of the E-cadherin, N-cadherin and Snail proteins. Thus, NRBP2 played a crucial role in the EMT process of BC cells.

### Overexpression of NRBP2 Decreases the Lung Metastasis of BC Cells *in vivo*

**Figure 5:**
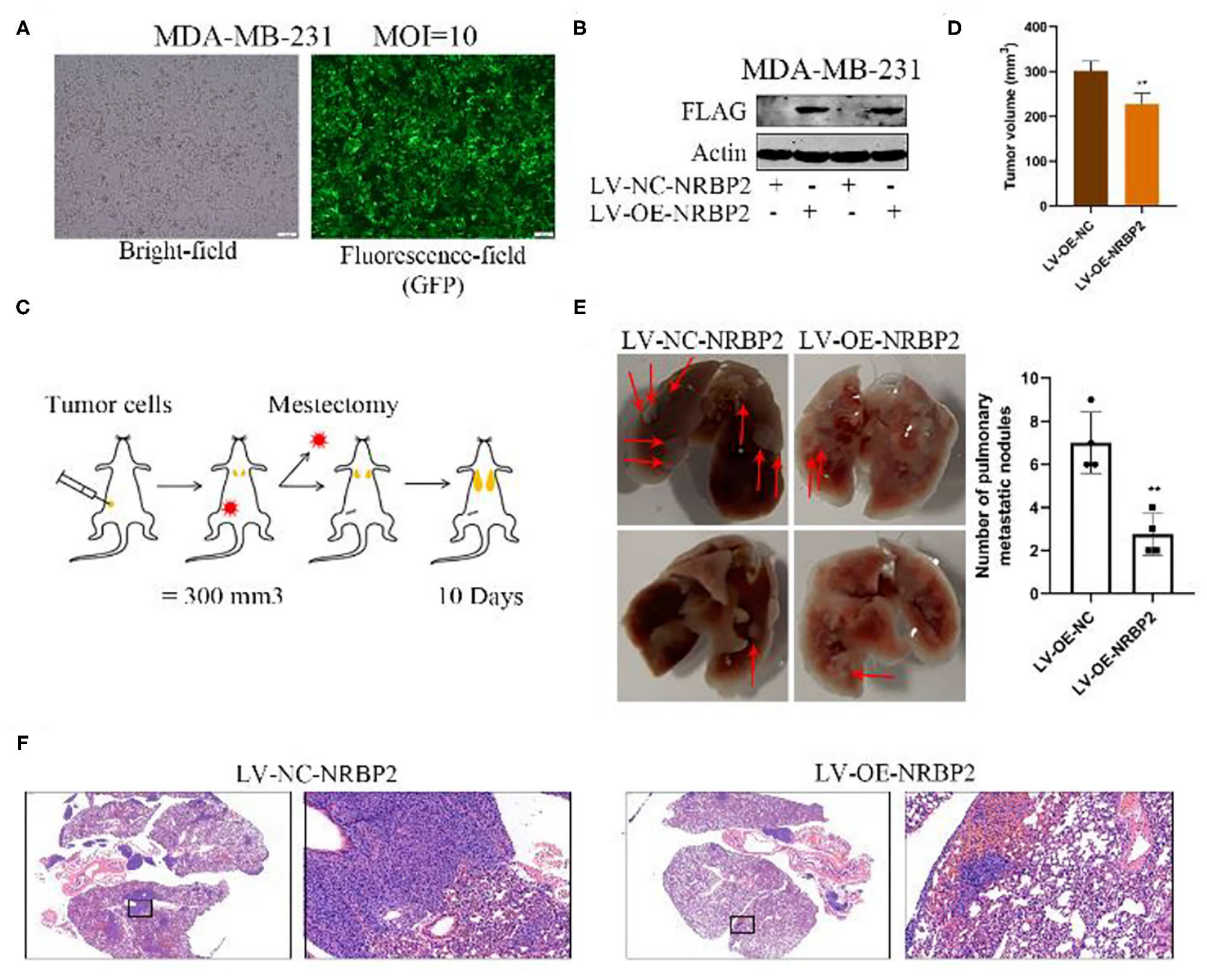
Overexpression of NRBP2 decreased the lung metastasis of BC cells *in vivo*. **(A)** The transfection efficiency of the NRBP2 overexpression plasmid in MDA-MB-231 cells. **(B)** The efficiency of NRBP2 overexpression in MDA-MB-231 cells. **(C)** Schematic of the experimental procedure used to assess lung metastasis. **(D)** Comparison of tumor volume from various groups. **(E)** Bright field images of the lung metastases in the control group and overexpression group (left panel) and the quantification of the metastatic tumors (right) (*n*=4). **(F)** HE staining of lung metastatic tumors. (magnification ×200) ^**^p < 0.01 compared with the control group.

We next performed a series of experiments *in vivo* to further verify the effect of NRBP2 on the EMT of BC cells observed *in vitro*. MDA-MB-231 cells stably overexpressing NRBP2 were successfully established to generate a mouse BC *in situ* model and detect lung metastasis (Figures 5A,B). As shown in Figures 5C,D, the MDA-MB-231 cells from each treatment group were injected into the mammary fat pad of mice (*n*=4). When the tumor size reached approximately 300 mm^3^, a mastectomy was performed. Then, the degree of lung metastasis in each group was observed after 10 days. Bright field images revealed more lung metastases in the LV-NC-NRBP2 groups than in the LV-OE-NRBP2 groups (Figure 5E). Moreover, consistent with images of real lung tissues, HE staining showed the same trend for lung metastasis, as measured by the size and number of nodules in stained lung tissue (Figure 5F). Based on these data, NRBP2 inhibited BC metastasis *in vivo*.

### NRBP2 Inhibits the EMT Through AMPK/mTOR Signaling

**Figure 6:**
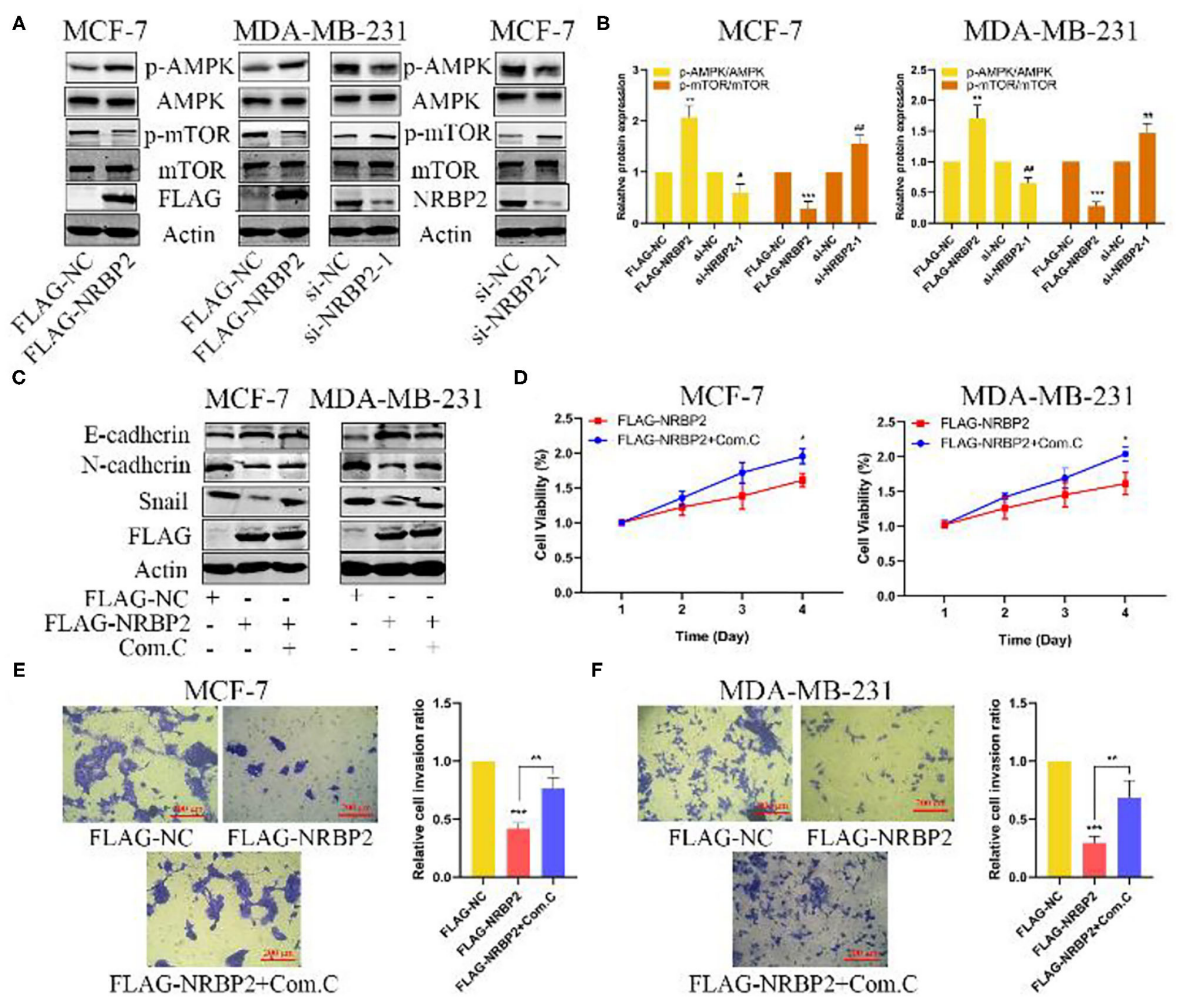
NRBP2 regulated the EMT of BC cells via AMPK signaling. **(A)** Levels of the p-AMPK, AMPK, p-mTOR and mTOR proteins in NRBP2-knockdown and -overexpressing cells were detected using Western blotting. **(B)** Quantitative analysis of the optical density ratio of p-AMPK, AMPK, p-mTOR and mTOR to β-Actin. **(C)** The levels of EMT-related proteins in NRBP2-overexpressing cells treated with Com.C (10 μM, 24 h) were detected using Western blotting. **(D)** Cell viability was measured using the CCK-8 assay in BC cells treated as described above. **(E,F)** Transwell assays were used to detect the invasion of two BC cell lines treated as described above. (magnification ×200) Right: Quantitative analysis of the percentage of invading cells. The values are presented as the means ± SD from three independent experiments. ^#^*p* < 0.05, ^##^*p* < 0.01, ^*^p < 0.05, ^**^p < 0.01 and ^***^p < 0.001 compared with the corresponding group.

**Figure 7:**
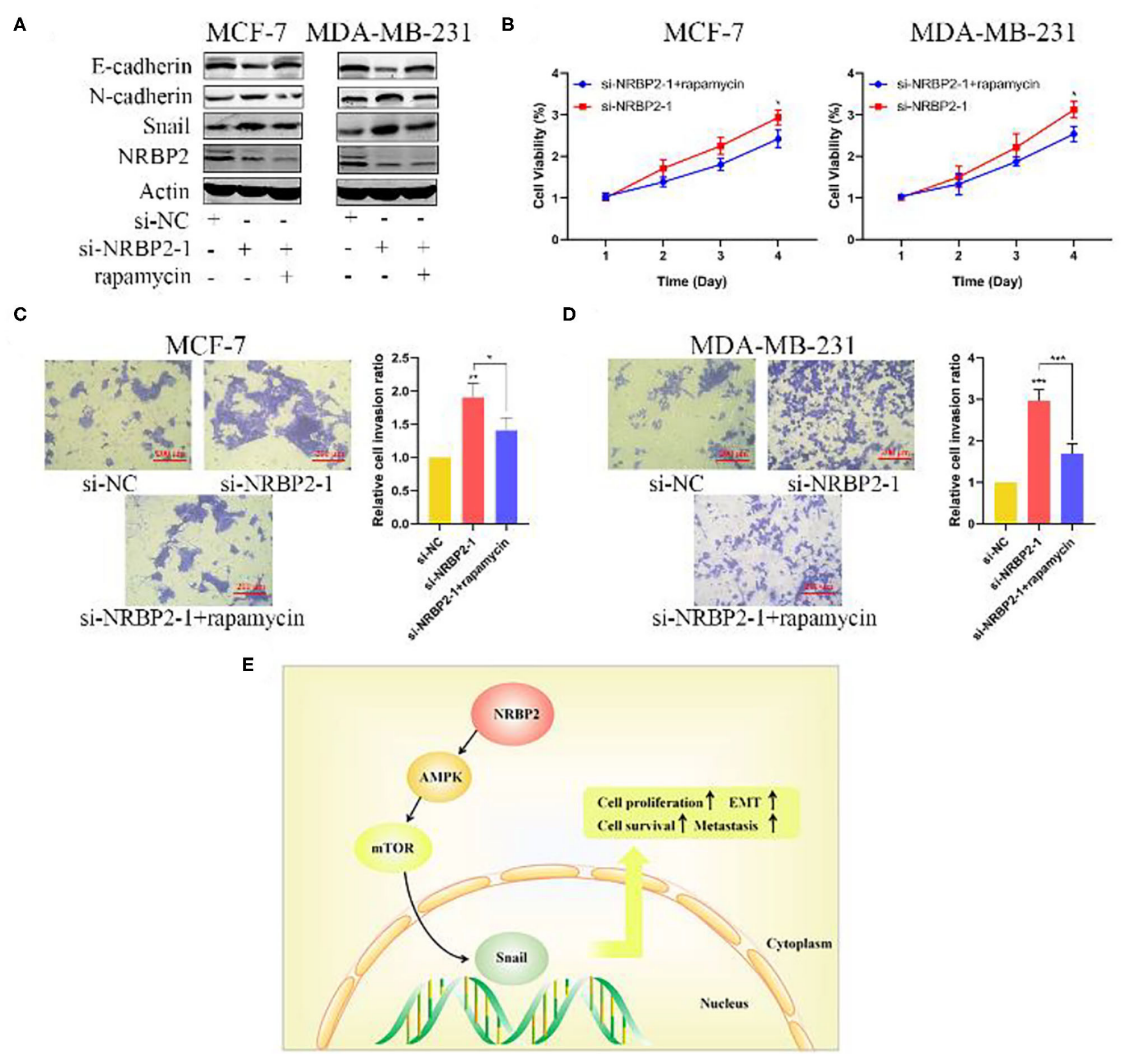
NRBP2 regulated the EMT in BC cells via AMPK/mTOR signaling. **(A)** The levels of EMT-related proteins in NRBP2-silenced cells treated with Com.C (1 μM, 24 h) were detected using Western blotting. **(B)** Cell viability was measured using the CCK-8 assay as described above. **(C,D)** The Transwell assay revealed the invasion of BC cells treated as described above. (magnification ×200) Right: Quantitative analysis of the percentage of invading cells. **(E)** Schematic model of the mechanism by which NRBP2 inhibits the progression of BC. The values are presented as the means ± SD from three independent experiments. ^*^p < 0.05, ^**^p < 0.01 and ^***^p < 0.001 compared with the corresponding group.

Based on an increasing number of studies, the AMPK/mTOR signaling pathway plays a vital role in tumor proliferation and metastasis. We next explored whether this signaling pathway was involved in the mechanism by which NRBP2 induced the EMT, proliferation and invasion of BC cells. Western blotting was used to detect the levels of p-AMPK, AMPK, p-mTOR and mTOR in MCF7 and MDA-MB-231 cells. The levels of p-AMPK were increased in NRBP2-overexpressing cells and decreased in NRBP2-knockdown cells. Correspondingly, compared with the changes in the levels of the p-AMPK protein, the level of p-mTOR showed the opposite trend (Figures 6A,B and Supplementary Figure 1E). AMPK signaling was blocked by its inhibitor Compound C (Com. C) to further examine the involvement of the AMPK/mTOR pathway in the process by which NRBP2 regulated the EMT. In NRBP2-overexpressing BC cells, the Com.C treatment decreased levels of the E-cadherin protein to some extent and increased the levels of N-cadherin and Snail (Figure 6C). In addition, the percentages of proliferating and invading cells were significantly increased after the Com.C treatment of NRBP2-overexpressing cells, indicating that AMPK inhibition partially rescued the inhibitory effect of NRBP2 overexpression on EMT progression, cell proliferation and invasion (Figures 6D–F). Similarly, in NRBP2-knockdown cells, rapamycin was used to suppress p-mTOR activation, and increased levels of the E-cadherin protein and decreased levels of N-cadherin and Snail were observed (Figure 7A and Supplementary Figure 1D). Furthermore, mTOR inhibition suppressed the proliferation and invasion of NRBP2-silenced cells (Figures 7B–D and Supplementary Figures 1B,C). Overall, we concluded that NRBP2 inhibited cell growth, invasion and the EMT via the AMPK/mTOR signaling pathway (Figure 7E).

## Discussion

Currently, the malignant progression of BC has been threatening the health of women worldwide and seriously affects the quality of life of patients. Therefore, current research is still focused on exploring potential therapeutic targets for inhibiting BC progression. NRBP2 is closely related to the development and differentiation of the brain. However, the exact role of NRBP2 in BC and the underlying specific molecular mechanism have not been reported. In our study, we clarified the role of NRBP2 in the occurrence and development of breast cancer for the first time. NRBP2 was expressed at lower levels in the tumor tissues than in normal tissues, and NRBP2 was related to the stage, type and lymph node metastasis of breast cancer. More interestingly, BC tissues with low NRBP2 expression not only contributed to the poor prognosis of all patients but also patients with different types of BC, such as Luminal A, Luminal B, Her+ and other subtypes. Additionally, NRBP2 overexpression inhibited the malignancy of BC cells by decreasing their proliferation and invasion and inhibiting the EMT process *in vitro* and BC lung metastasis *in vivo*. In contrast, NRBP2 knockdown exerted the opposite effects. Finally, NRBP2 regulated BC cell proliferation, invasion and the EMT by activating the AMPK/mTOR signaling pathway, suggesting that this pathway may be a downstream target of NRBP2. Accordingly, NRBP2 functioned as a tumor suppressor in BC, and had irreplaceable value in the control of BC progression.

NRBP2, a gene identified through a mammalian gene collection program (15), was shown to be dramatically upregulated at the transcriptional level during the differentiation of NSPCs (16). In addition, NRBP2 is expressed in the hippocampus and ventricular wall of the embryonic mouse brain, whereas it is mainly located in hippocampal neurons and Purkinje cells in the adult brain (9). Based on emerging evidence, NRBP2 participates in the progression of various tumors by inhibiting the proliferation and metastasis of tumor cells and promoting tumor cell apoptosis (10, 11). As shown in the present study, NRBP2 overexpression markedly increased cell proliferation and invasion *in vitro*, and promoted the lung metastasis of BC cells *in vivo*. In contrast, knockdown of NRBP2 exerted opposite effects on cell proliferation and invasion *in vitro*. Furthermore, the RFS and OS curves of patients with various types of BC indicated that the prognosis of patients with BC presenting high NRBP2 expression was better than patients with BC presenting low NRBP2 expression. More interestingly, consistent with previously reported results, our IHC results also revealed that NRBP2 was located in the cytoplasm of normal breast tissues and BC tissues. Based on these findings, NRBP2 functioned as a tumor suppressor gene and novel prognostic factor in BC.

Tumor proliferation, invasion and metastasis are components of an extremely complex dynamic cascade of molecular processes (17). In the epithelial-mesenchymal transition (EMT), epithelial cells lose cell-to-cell connections and acquire a fibroblast-like morphology, which has been proven to be the first step in the cascade reaction process (18). Tumor cells undergoing the EMT usually present a loss of epithelial markers (E-cadherin) and gain of mesenchymal markers (N-cadherin) (19). The EMT renders tumor cells highly invasive, leading to the distant metastasis of tumors (20, 21). In our study, NRBP2 overexpression significantly suppressed the EMT phenotype in BC cells, while NRBP2 knockdown exerted the opposite effect on EMT-related proteins. Additionally, in NRBP2-overexpressing BC cells, knockdown of NRBP2 partially restored the levels of EMT-related proteins. Therefore, NRBP2 attenuates the malignant progression of BC by inhibiting the EMT in BC cells.

Most tumors are often accompanied by abnormal activation or inhibition of various molecules and signaling pathways during their development and metastasis (22, 23), and AMPK/mTOR signaling is one of the irreplaceable pathways in tumor cells (24) because it not only regulates the level of intracellular autophagy (25) but also regulates biological processes such as the proliferation, apoptosis and invasion of tumor cells (25). AMPK signaling plays a critical role in the development of almost all cancer cells, and its mechanism is to regulate tumor progression through a variety of downstream effector molecules or pathways, such as the mTOR complex (26). AMPK inhibits the phosphorylation of the mTOR complex and its downstream molecules through various molecular mechanisms, thereby reducing the progression and metastasis of tumor cells (27). In our study, NRBP2 regulated the activation of the AMPK/mTOR pathway in BC cells. Next, we used AMPK inhibitors and mTOR inhibitors to explore whether the AMPK/MTOR pathway played a role in the inhibition of cell proliferation and invasion induced by NRBP2. Com.C and rapamycin partially reversed the changes in cellular biological processes and the EMT phenotype after NRBP2 overexpression and knockdown, respectively. Therefore, NRBP2 may modify BC cell growth, invasion and the EMT by regulating the AMPK/MTOR pathway.

In summary, we have described the role and molecular mechanisms of NRBP2 in BC for the first time. NRBP2 was downregulated in BC tissues compared to normal breast tissues, and decreased NRBP2 expression levels contributed to the poor prognosis of patients with BC. Mechanistically, NRBP2 modulated cell proliferation, invasion and the EMT process by regulating the AMPK/MTOR pathway. The present study not only reveals the important predictive role of NRBP2 in the prognosis and malignant progression of BC but also provides a new theoretical basis and potential therapeutic target for the treatment of BC.

However, more studies are still needed to further explore the tumor suppressor role of NRBP2 in the malignant progression of BC, such as the specific localization of NRBP2 in BC cells, the possibility of NRBP2 translocation to the nucleus, and the specific mechanism by which NRBP2 regulates the AMPK/mTOR pathway, among other processes. After elucidating its roles, we will obtain a better understanding of the tumor suppressor properties of NRBP2 and lay a theoretical foundation for NRBP2 to become a potential emerging therapeutic target for tumor treatment.

## Data Availability Statement

The original contributions presented in the study are included in the article/Supplementary Material, further inquiries can be directed to the corresponding author/s.

## Ethics Statement

The studies involving human participants were reviewed and approved by the Ethics Committee of Renmin Hospital of Wuhan University. The patients/participants provided their written informed consent to participate in this study. The animal study was reviewed and approved by Animal Committee of Wuhan University.

## Author Contributions

ZL, BL, and CL carried out experiments and wrote the manuscript. SiS and HZ took on the statistical analysis. ShS, ZW, and XZ designed the study. All authors contributed to the article and approved the submitted version.

## Conflict of Interest

The authors declare that the research was conducted in the absence of any commercial or financial relationships that could be construed as a potential conflict of interest.
