# Supplementary material for: NRBP2 Functions as a Tumor Suppressor and Inhibits Epithelial-to-Mesenchymal Transition in Breast Cancer
Source: Front Oncol. 2021 Mar 18;11:634026. doi: 10.3389/fonc.2021.634026 (PMC8012753; doi:10.3389/fonc.2021.634026)
Supplement: Supplementary file 1 [file Image_1.pdf]

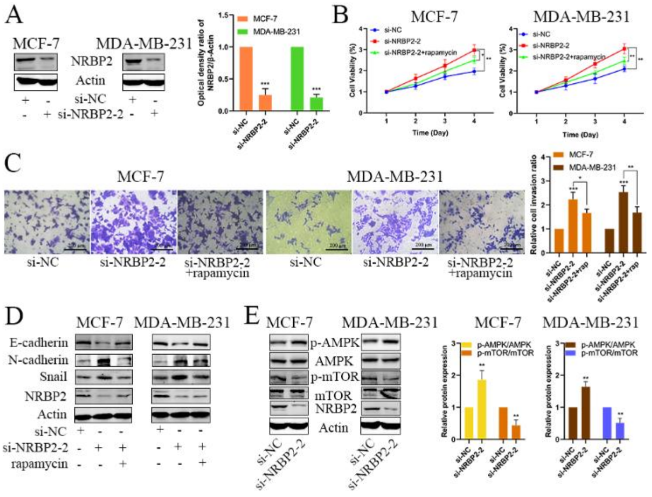

Supplementary Figure 1. Knockdown of NRBP2 promotes BC cell biological functions and AMPK/mTOR signaling pathway activation. (A) Cell viability was measured using the CCK-8 assay in NRBP2-silenced cells treated with or without Com.C (1 μM, 24 h). (C) The Transwell assay revealed the invasion of BC cells treated as described above. Right panel: Quantitative analysis of the percentage of invading cells. (D) The levels of EMT-related proteins of two BC cell lines treated as described above were detected using Western blotting. (E) Levels of the p-AMPK, AMPK, p-mTOR and mTOR proteins in NRBP2-knockdown cells were detected using Western blotting. *p<0.05, **p<0.01 and ***p<0.001 compared with the corresponding group.
